# Ultrasound microbubble-mediated transfection of NF-κB decoy oligodeoxynucleotide into gingival tissues inhibits periodontitis in rats in vivo

**DOI:** 10.1371/journal.pone.0186264

**Published:** 2017-11-01

**Authors:** Hiroyuki Yamaguchi, Yuji Ishida, Jun Hosomichi, Jun-ichi Suzuki, Kasumi Hatano, Risa Usumi-Fujita, Yasuhiro Shimizu, Sawa Kaneko, Takashi Ono

**Affiliations:** 1 Department of Orthodontic Science, Graduate School of Medical and Dental Sciences, Tokyo Medical and Dental University, Tokyo, Japan; 2 Department of Advanced Clinical Science and Therapeutics, Graduate School of Medicine, The University of Tokyo, Tokyo, Japan; Charles P. Darby Children's Research Institute, UNITED STATES

## Abstract

Periodontitis is a chronic infectious disease for which the fundamental treatment is to reduce the load of subgingival pathogenic bacteria by debridement. However, previous investigators attempted to implement a nuclear factor kappa B (NF-κB) decoy oligodeoxynucleotide (ODN) as a suppressor of periodontitis progression. Although we recently reported the effectiveness of the ultrasound-microbubble method as a tool for transfecting the NF-κB decoy ODN into healthy rodent gingival tissue, this technique has not yet been applied to the pathological gingiva of periodontitis animal models. Therefore, the aim of this study was to investigate the effectiveness of the technique in transfecting the NF-κB decoy ODN into rats with ligature-induced periodontitis. Micro computed tomography (micro-CT) analysis demonstrated a significant reduction in alveolar bone loss following treatment with the NF-κB decoy ODN in the experimental group. RT-PCR showed that NF-κB decoy ODN treatment resulted in significantly reduced expression of inflammatory cytokine transcripts within rat gingival tissues. Thus, we established a transcutaneous transfection model of NF-κB decoy ODN treatment of periodontal tissues using the ultrasound-microbubble technique. Our findings suggest that the NF-κB decoy ODN could be used as a significant suppressor of gingival inflammation and periodontal disease progression.

## Introduction

Periodontitis is a chronic infectious disease that is caused by the accumulation of bacteria, and it leads to the destruction of the surrounding periodontal structure, including pocket deepening, attachment loss, and alveolar bone loss [1]. The fundamental treatment for periodontitis is still to reduce the load of subgingival pathogenic bacteria by instrumental debridement via surgical or non-surgical approaches [2]. However, complete removal of pathogenic biofilms is difficult, as some pathogens are embedded in soft tissues and/or located in anatomically inaccessible areas. Therefore, antibiotics or antiseptics are sometimes applied as adjuvant treatments for periodontal infection in combination with mechanical instrumentation [3].

Nuclear factor kappa B (NF-κB) is a common signaling molecule involved in many types of inflammation, and in particular, is known to play an important role in the initiation of immune and inflammatory reactions in periodontal tissues [4]. NF-κB was the first transcription factor found to bind a DNA element in a kappa immunoglobulin light-chain enhancer [5]. The expression and activation of NF-κB initiates a downstream signaling cascade involving various inflammatory cytokines, including interleukin-1β (IL-1β) and tumor necrosis factor-α (TNF-α), as well as several adhesion molecules, such as intercellular adhesion molecule-1 (ICAM-1) [6]. Receptor activator NF-κB ligand (RANKL) is widely recognized as a key factor involved in osteoclastogenesis and as a regulator of NF-κB activation [7]. Several previous studies suggested that transfection of target cells and tissues with a NF-κB decoy oligodeoxynucleotide (ODN), which has a sequence similar to the NF-κB DNA binding site and selectively blocks NF-κB activation, is an efficient method for suppressing NF-κB function [8, 9]. Indeed, previous studies have shown that this decoy ODN successfully suppresses the symptoms of various inflammatory and autoimmune diseases, including atopic dermatitis and immunorejection [10–12].

Of particular interest is the methodology used for transfecting cells or tissues with the decoy ODN, which often includes the use of ointments and injections. Recently, however, a new method for decoy ODN transfection was proposed involving ultrasound and microbubbles [13–16]. Microbubbles have the potential to be highly proficient drug/gene delivery devices, as they create small holes on the cell surface, allowing for easy and rapid gene transfection and drug delivery [17, 18]. Specifically, it appears that the core of the cavitation of the microbubble is altered by ultrasound stimulation, resulting in more holes being made in cells close to the microbubble compared with those created in experiments conducted without ultrasound stimulation. Suzuki et al. and Inagaki et al. successfully utilized this method to transfect arterial tissues with decoy ODN and to investigate the resulting suppressive effects of this decoy on target gene expression [19, 20]. Similarly, we recently demonstrated the effectiveness of the ultrasound-microbubble approach as a tool for transfecting the NF-κB decoy ODN into healthy rodent gingival tissue in vivo [21]. However, the ultrasound-microbubble technique has yet to be applied to the treatment of periodontitis in animal models.

The objective of this study was to investigate whether transfection of the NF-κB decoy ODN via the ultrasound-microbubble technique could effectively prevent gingival inflammation and alveolar bone loss in a rat model of ligature-induced periodontitis.

## Materials and methods

### Animals

In total, 36 six-week-old male Wistar/ST rats (Sankyo-lab, Tokyo, Japan) were randomly assigned to three groups (n = 12 each): control, P (periodontitis model), and PUM (periodontitis model with ultrasound-microbubble-mediated application of NF-κB decoy ODN). All treatments were performed following anesthetization via intraperitoneal injection of 6% pentobarbital sodium (10 ml/kg) (Somnopentyl, Kyoritsuseiyaku, Tokyo, Japan). All animal experiments were approved by the Institutional Animal Care and Use Committee of Tokyo Medical and Dental University (#0160308A, #0170210A).

### NF-κB decoy ODN transfection

Transfection of NF-κB decoy ODN was performed as previously described [21]. The phosphorothioate NF-κB decoy ODN sequences utilized were as follows: 5'-CCTTGAAGGGATTTCCCTCC-3' and 3'-GGAGGGAAATCCCTTCAAGG-5'. For irradiation, a Sonitron 2000 ultrasound machine (Nepa Gene, Tokyo, Japan) equipped with a 3.0 mm (diameter) probe (Nepa Gene) was utilized, according to the manufacturer’s instructions [22]. For this procedure, 90 μl of the NF-κB decoy (10 μg) was added to 20 μl of the microbubbles (SV-25; Nepa Gene), and the mixture was suspended in 90 μl of echo gel.

**Fig 1:**
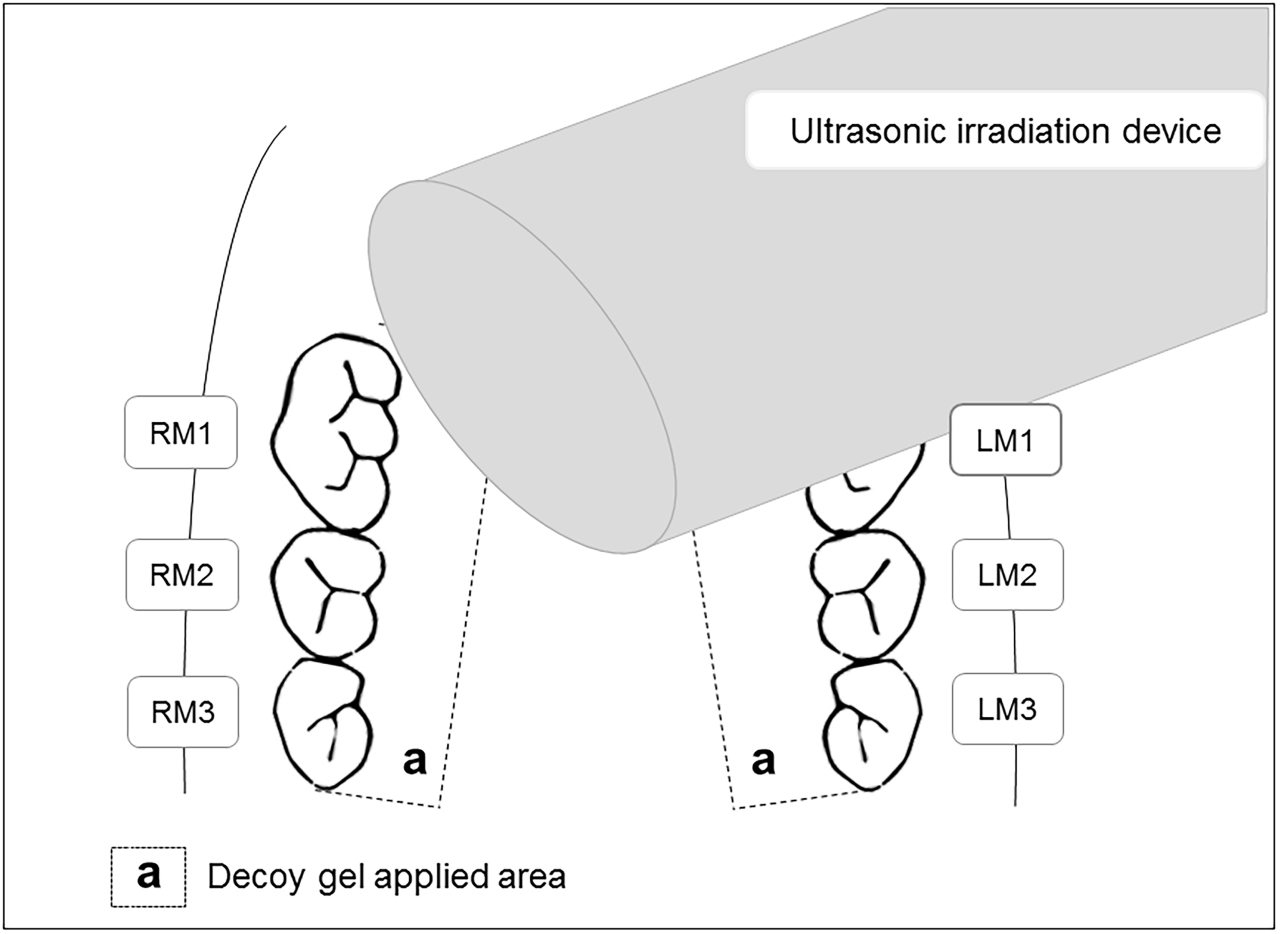
Schematic illustration of the ultrasound-microbubble method for transfection of palatal gingival tissues. Immediately after application of the decoy gel to the tissue area (a) comprising the palatal gingival tissue from the first maxillary molar (M1) to the third maxillary molar (M3), gingival tissues were transfected with the NF-κB decoy oligodeoxynucleotide (ODN) using a Sonitron 2000 device.

To generate the periodontitis model, rats were allowed to adapt to laboratory conditions for 1 week, after which a sterilized 5–0 silk ligature was tied around the cervix of each bilateral maxillary second molar of the rats in the P and PUM groups, as previously described [23]. To inhibit periodontitis progression, the maxillary gingiva of the rats in the PUM group were transfected with the NF-κB decoy ODN via the ultrasound-microbubble technique every 2 days for 2 weeks in accordance with a previous study [24]. After ligation, NF-κB decoy gel was applied to the palatal gingiva on both sides of the rat maxillae. Next, ultrasound radiation was immediately applied to the gingiva of the animals in the PUM group (Fig 1). All rats in this group were maintained without any food or water for 2 hours after radiation to increase the effectiveness of the inoculation. All rats were sacrificed via a carbon dioxide stunning method at 7 or 14 days after ligation, and the maxillae of each rat were dissected and analyzed as described below.

### Micro-CT analyses

**Fig 2:**
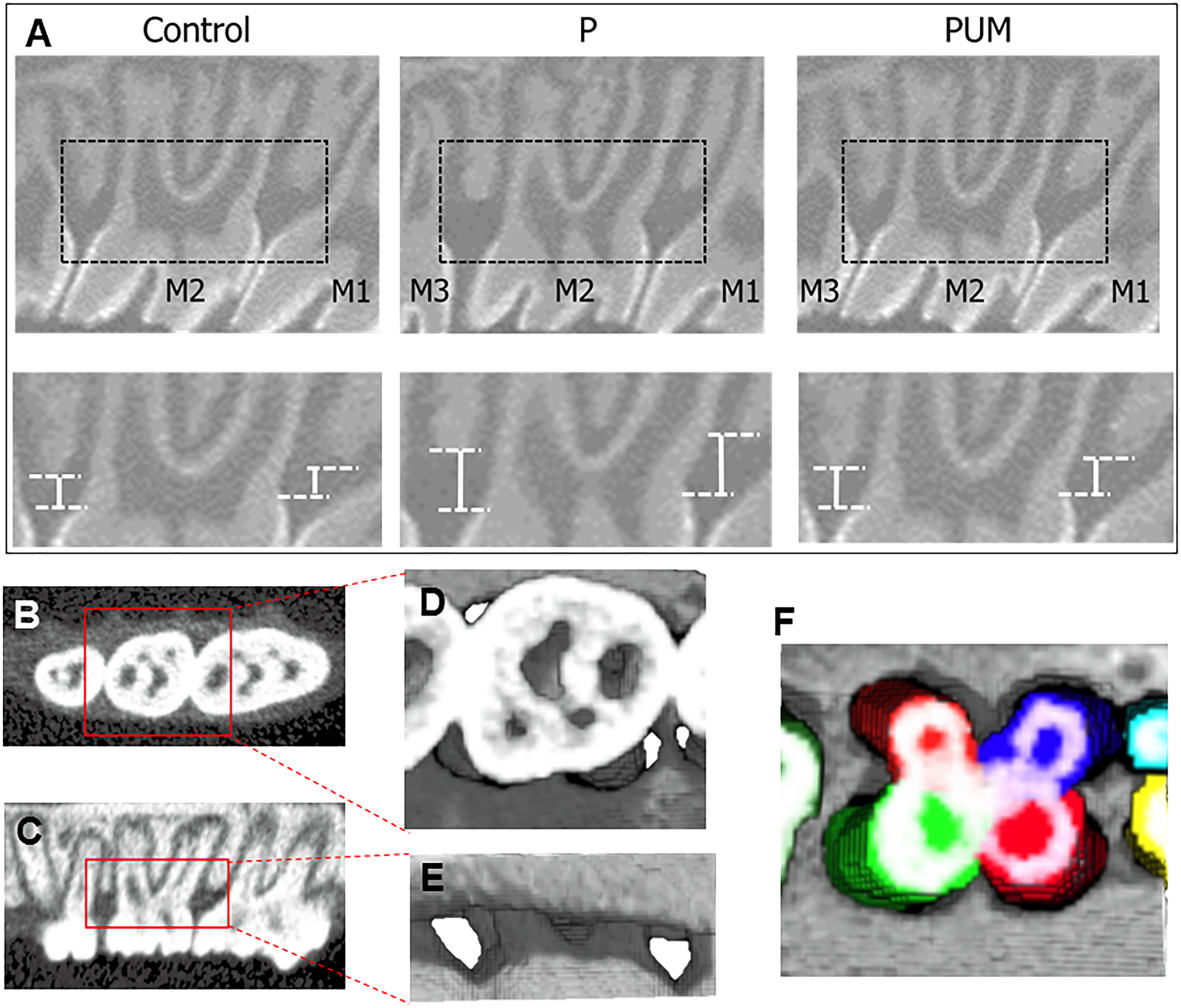
Depiction of the method used for micro-computed tomography (micro-CT) analysis of rat molars. **(A)** Linear measurements were taken of alveolar bone loss in the interdental space from the cement enamel junction (CEJ) to the alveolar bone crest (ABC). **(B)** Representative depiction of the region of interest (ROI), which comprised a rectangular area drawn 0.33 mm axially from the tooth crown in each direction. **(C)** Representative ROI including, vertically, the coronal halves of the slices through the mesial root apex to the CEJ. **(D and E)** Representative three-dimensional (3D) images of the volume of interest (VOI): **(D)** horizontal view **(E)** sagittal view. **(F)** Representative volumetric measurement obtained using the 3D-generated ROI. Colored regions (blue: mesial palatal root of M2, orange: distal palatal root of M2, red: mesial buccal root of M2, yellow-green: distal buccal root of M2, light blue and yellow: distal root of M1, green: mesial root of M3) indicate tooth parts that were removed from the VOI for measurement of the alveolar bone volume.

Alveolar bone morphology and quality were analyzed by cone-beam micro-computed tomography using an SMX-100CT system (Shimadzu, Kyoto, Japan) and 3D trabecular bone analysis software (TRI/3D-BON; RATOC System Engineering Co., Tokyo, Japan). After scanning of the alveolar bone around the maxillary second molar, three-dimensional microstructural image data were reconstructed, and structural indices were calculated with TRI/3D-BON software. The distances between the alveolar bone crest (ABC) and the cement enamel junction (CEJ) in the mesiodistal direction of the maxillary second molars were also determined using TRI/3D-BON software (Fig 2A) [25]. Average ABC–CEJ distances were used to evaluate the amount of alveolar bone loss. The cross-sectional image was defined such that the mesial palatal root and distal palatal root of M2 were parallel at CEJ height.

To analyze the volume of alveolar bone around the maxillary second molar on day 14 after ligation, we assessed the bone volume using a previously described method [26]. For calibration, the region of interest (ROI) was determined according to the size of the second molar, rather than forming a fixed ROI for each sample, in consideration of differences in the sizes of the maxillary second molars. Axially, a rectangle that was 0.33 mm from the tooth crown in each direction was set as the ROI (Fig 2B and 2D). Vertically, coronal halves of the slices through the mesial root apex to the CEJ were included in the volume of interest (VOI) (Fig 2C and 2E). Therefore, a standard VOI suitable for tooth size was formed for each second molar. Tooth parts were removed from the VOI, and the remaining bone volume of the VOI was recorded in mm^3^ (Fig 2F).

### Microdissection and quantitative PCR analyses

Frozen non-decalcified sections were prepared for histological investigation using a cryofilm-transfer kit (Finetec, Gunma, Japan), as previously described. The maxillae isolated as described above were frozen by quenching in cold hexane, embedded in 5% SCEM (super cryoembedding medium) gel, and further frozen in cold hexane. The frozen SCEM samples were then sliced frontally with disposable carbide tungsten steel blades (Leica Microsystems, Wetzlar, Germany). The trimmed surface was covered with an adhesive film (Finetec, Gunma, Japan), and each sample was serially sectioned frontally along with the film at a thickness of 10 μm. For histological analysis of periodontal tissue, the sections were stained with hematoxylin and eosin (H&E) (Leica Microsystems) and observed with an optical microscope (ECLIPSE 80i; Nikon, Tokyo, Japan). The gingival tissue lacking keratinized epithelium layer in each section was identified based on HE staining and was collected from the sections with an LMD7000 laser microdissection apparatus (Leica Microsystems) [27].

Total RNA was extracted from gingival tissues using a RecoverAll Total Nucleic Acid Isolation Kit (Thermo Fisher Scientific, Waltham, MA, USA). Complementary DNA was synthesized from total RNA via reverse transcription with random primers using PrimeScript RT Reagent Kit (Takara Bio, Shiga, Japan). Quantitative PCR assays were performed in triplicate for each sample using a 7500 Real-Time PCR System (Applied Biosystems, Foster City, CA, USA). PCR analyses were conducted using gene-specific primers and fluorescently labeled TaqMan probes (Takara Bio). Appropriate primers were chosen for real-time PCR amplification of genes encoding IL-1β, TNF-α, ICAM-1, RANKL, and Hprt-1. The thermocycling conditions used were as follows: 95°C for 30 s, followed by 40 cycles of 95°C for 5 s and 60°C for 34 s. Gene expression levels were calculated according to the ΔΔCt method of relative quantification. The threshold cycle (Ct) value for each target mRNA (*IL1B*, *TNFA*, *ICAM1*, or *RANKL*) was normalized to that of the internal control (*Hprt1*) in the same sample (ΔCt = Ct_target_−Ct_*Hprt1*_), followed by normalization to the control (ΔΔCt_P_ = ΔCt_Pgroup_−ΔCt_control_; ΔΔCt_PUM_ = ΔCt_PUMgroup_−ΔCt_control_). The fold change in expression was calculated as the relative quantification value (RQ; 2^−ΔΔCt^) [28].

### Statistical analysis

Statistical calculations were performed using statistical analysis software (IBM SPSS Statistics Version 20.0; SPSS Statistics, Inc., Chicago, IL, USA). After testing for normality and equal variance, intergroup comparisons were conducted via one-way analysis of variance (ANOVA) and Tukey’s post-hoc testing. Results are presented as the mean ± standard error (n = 6 each). Differences were considered to be significant at P < 0.05.

## Results

### Suppression of alveolar bone loss and gingival inflammation by NF-κB decoy ODN treatment via ultrasound-microbubble method according to micro-CT and histological analyses

**Fig 3:**
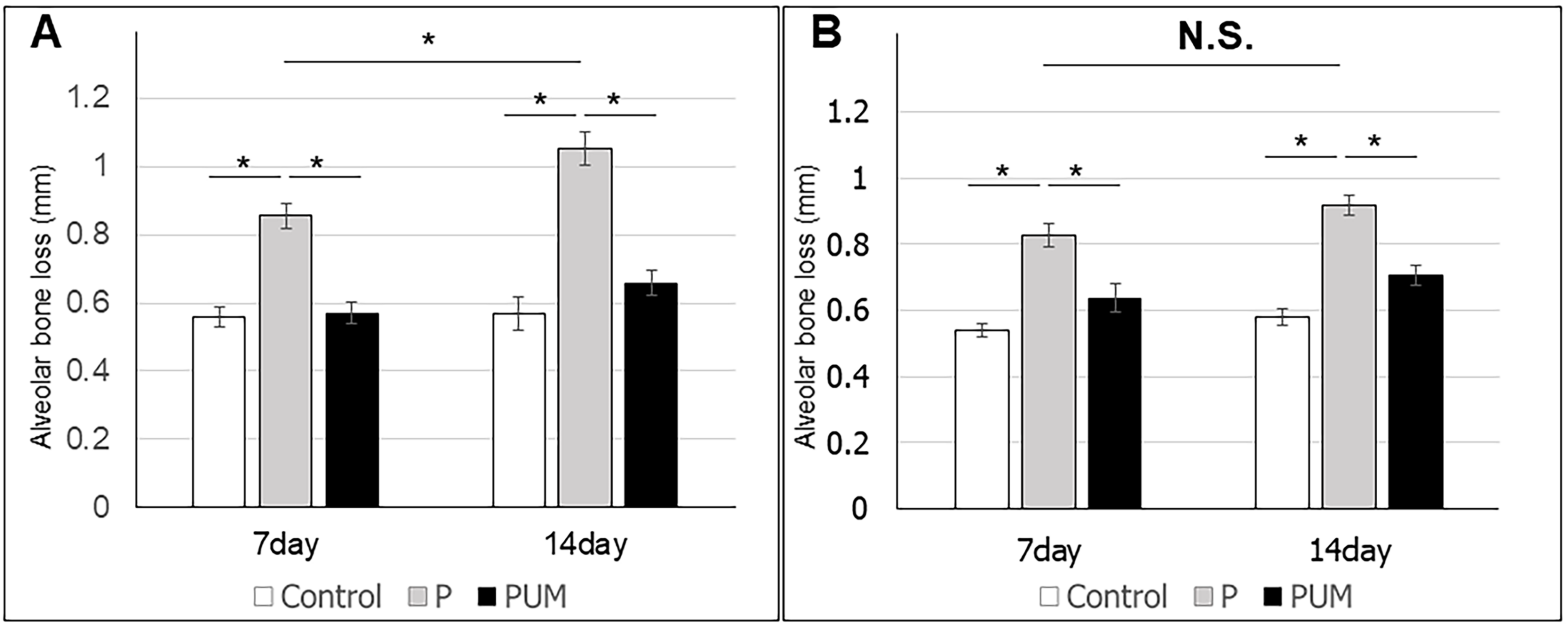
Alveolar bone loss in control, periodontitis (P), and periodontitis with ultrasound-microbubble-mediated application of NF-κB decoy oligodeoxynucleotide (ODN) (PUM) in rats. Graphic depictions of the mean alveolar bone loss (mm) between **(A)** the first (M1) and second (M2) maxillary molars and **(B)** M2 and the third maxillary molar (M3) in the rats in each group. Results are expressed as the mean ± standard error (n = 6) of the distance from the cement enamel junction (CEJ) to the alveolar bone crest (ABC. *P < 0.05; N.S., not significant).

**Fig 4:**
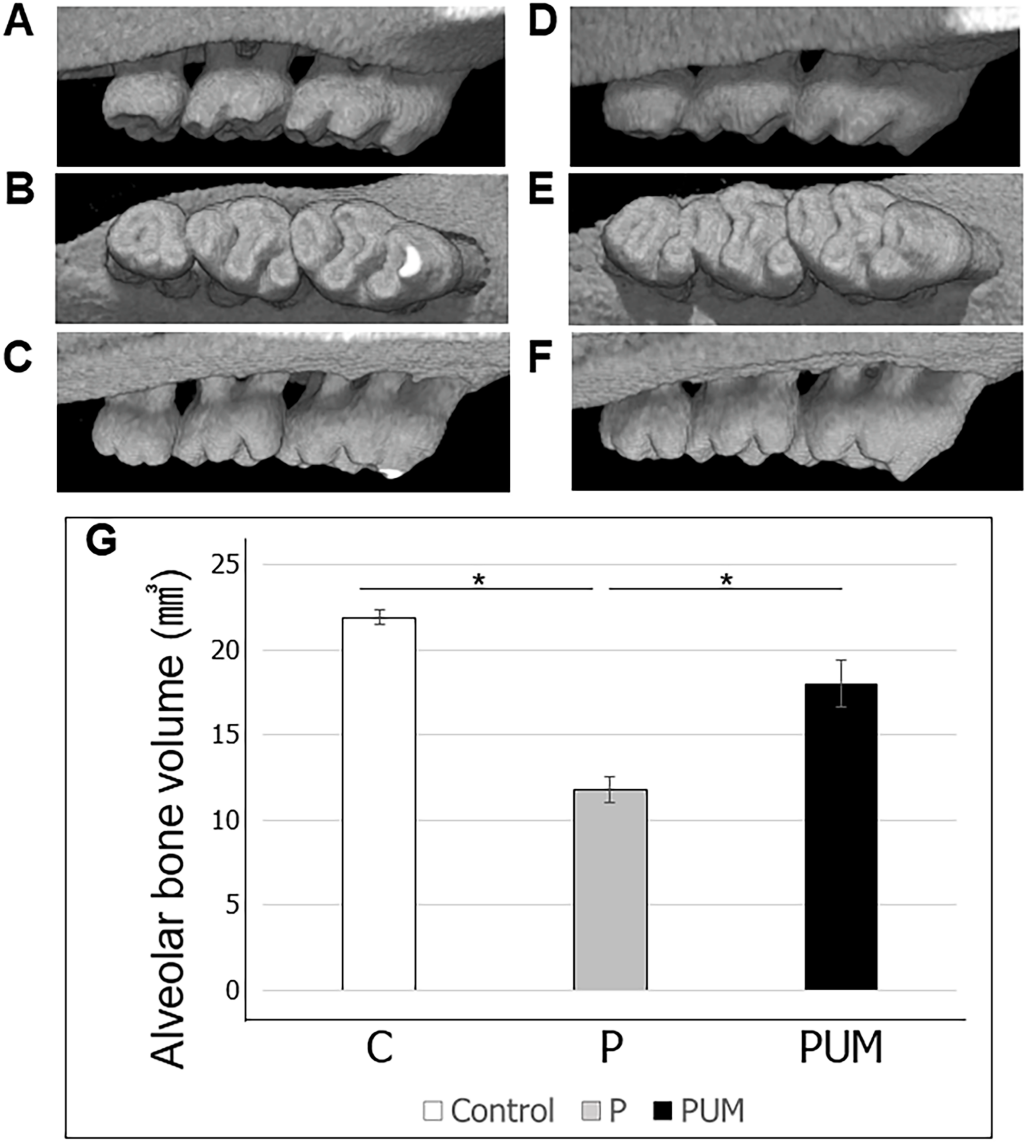
Three-dimensional analysis. (**A, B and C**) Three-dimensional images of rats with periodontitis (P); A: buccal side view, B: occlusal surface view, C: palatal side view. (**D, E and F**) Three-dimensional images of periodontitis with ultrasound-microbubble-mediated application of NF-κB decoy oligodeoxynucleotide (ODN) (PUM) in rats; D: buccal side view, E: occlusal surface view, F: palatal side view. **(G)** Alveolar bone volume in control, P, and PUM rats. Graphic depiction of alveolar bone volume (mm^3^) in the rats in each group. Results are expressed as the mean ± standard error (n = 6) of the volume of alveolar bone. *P < 0.05.

**Fig 5:**
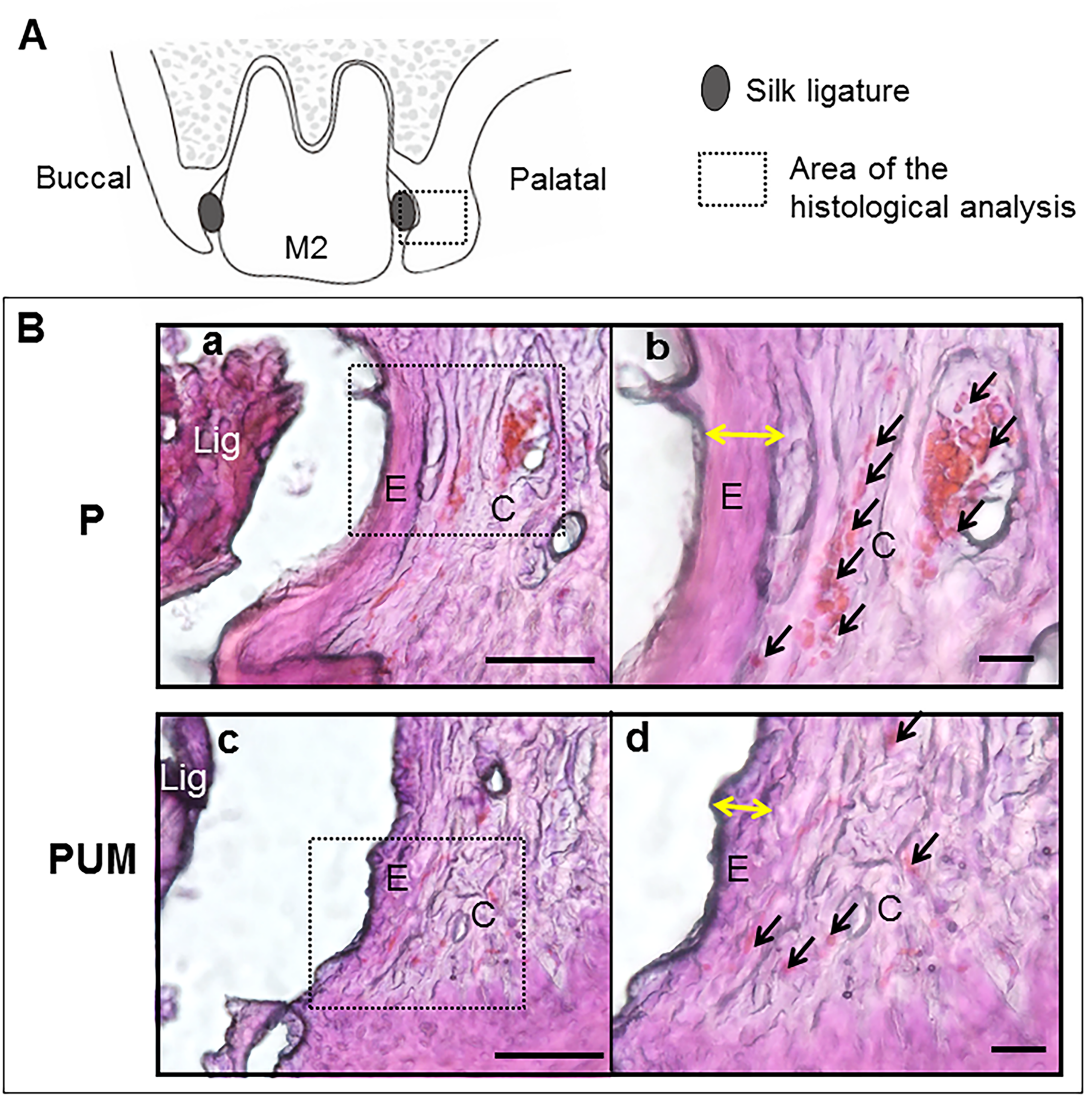
Histological images of the P and PUM groups. Comparative HE staining images taken at the M2 of representative rats in the P and PUM groups. The images on the right are magnified views of the inset in the corresponding left images. **(A)** Schematic illustration of these coronal sections in P and PUM groups. The inset in the schematic illustration indicates the area of the B images. (**B- a, b**) The periodontium of the P group rats showed increases in the capillaries (black arrows) and thickening of the junctional epithelium (yellow arrows). (**B- c, d**) The periodontium of the PUM rats showed decreased inflammatory response compared to the P group. Lig: silk ligature, E: junctional epithelium, C: gingival connective tissue. Bar = 100 μm.

The body weights of the rats in the three groups were statistically equivalent throughout the experimental period (refer to S1 Table). Subsequent micro-CT analysis of alveolar bone loss detected bone resorption, as evidenced by marked increases in the ABC–CEJ distances in the mesiodistal direction of the maxillary second molars, in the rats in the P group compared with those in the control group on days 7 and 14 after ligation (Fig 3A and 3B). Notably, this distance was reduced in the PUM group compared with that in the P group, and there was no significant difference in the ABC–CEJ distances of the control and PUM groups on days 7 and 14. In addition, the ABC–CEJ distance between M1 and M2 in the VOI on day 14 was significantly larger than that on day 7 in the P group. In contrast, the distances between M2 and M3 were statistically equivalent on days 7 and 14. The volume of alveolar bone around the maxillary second molar on day 14 after ligation was significantly smaller in the P group than in the control and PUM groups; there was no significant difference between the volumes in the control and PUM groups on day 14 after ligation (Fig 4). The histological features of the periodontium in the P group rats indicated some inflammatory responses such as vasodilation, increase of the blood cell components in the gingival connective tissue, and thickening of the junctional epithelial layer (Fig 5B-a and 5B-b), compared to that of the control group. In contrast, the histological features of the PUM group rats showed suppression of gingival inflammatory responses, comparing to that of the P group (Fig 5B-c and 5B-d). Additionally, no histological damage or abnormality was detected in the PUM group.

### Suppression of gingival expression of inflammatory cytokines by NF-κB decoy ODN treatment

**Fig 6:**
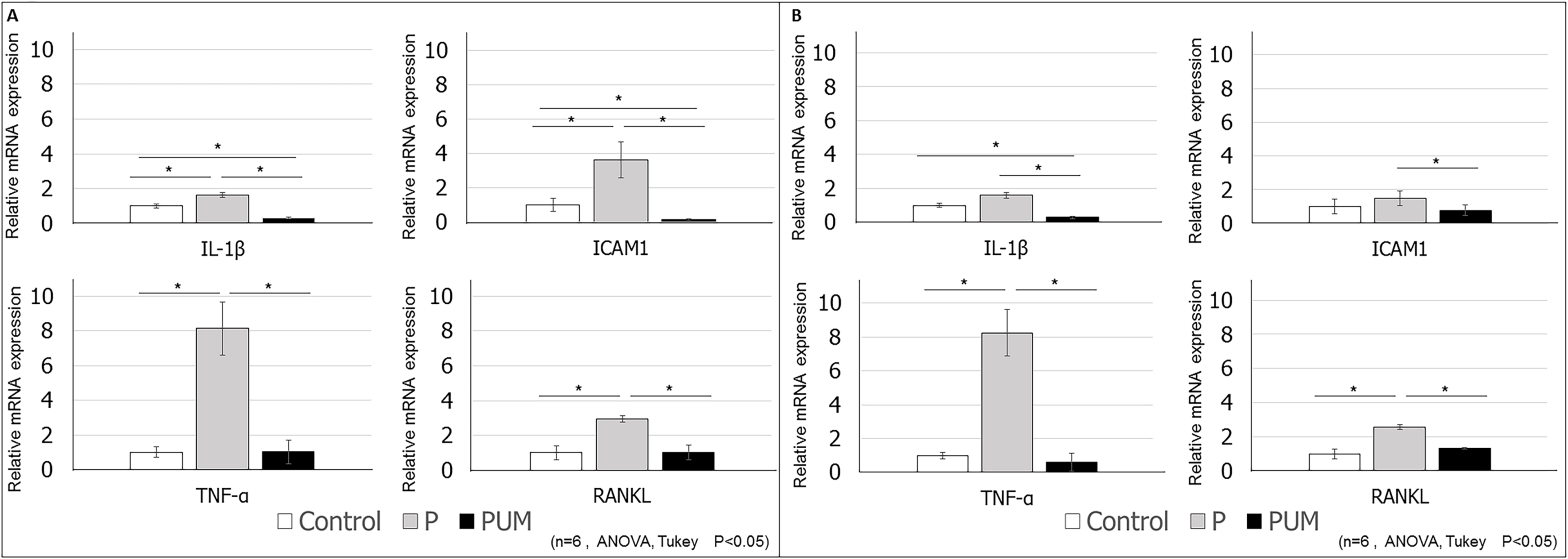
Evaluation of the expression of periodontitis markers of palatal gingiva in control, periodontitis (P), and periodontitis with ultrasound-microbubble-mediated application of NF-κB decoy oligodeoxynucleotide (ODN) (PUM) in rats. Relative expression levels of *IL1B*, *TNFα*, *ICAM1*, and *RANKL* within the palatal gingival tissues of rats in each group (n = 6 per group) were determined by RT-PCR analysis at **(A)** 7 days and **(B)** 14 days post-treatment. The mRNA expression levels in the control group were set to a value of 1. Data are presented as the mean ± standard deviation for each group. *P < 0.05.

**Fig 7:**
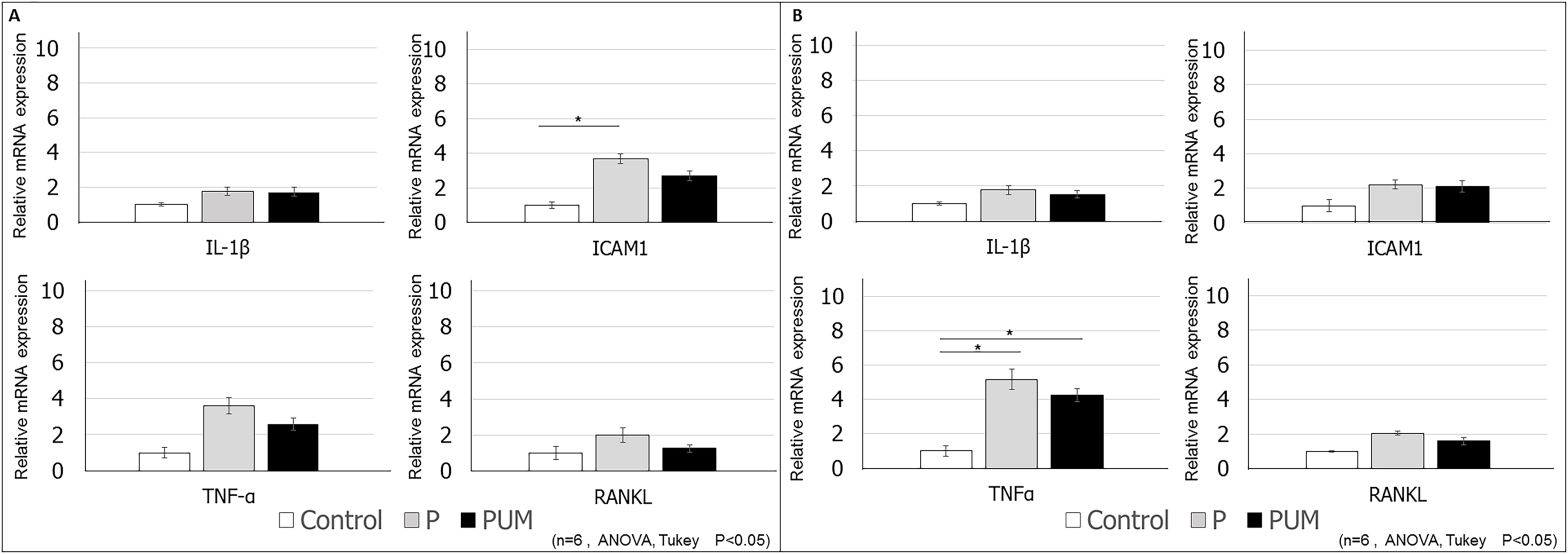
Evaluation of the expression levels of periodontitis markers in the buccal gingiva in control, periodontitis (P), and periodontitis with ultrasound-microbubble-mediated application of NF-κB decoy ODN (PUM) in rats. The relative expression levels of *IL1B*, *TNFα*, *ICAM1*, and *RANKL* within the buccal gingival tissues of rats in each group (n = 6 per group) were determined by RT-PCR analysis at **(A)** 7 days and **(B)** 14 days post-treatment. The mRNA expression levels in the control group were set to a value of 1. Data are presented as the mean ± standard deviation for each group. *P < 0.05.

On day 7 after ligation, the mRNA expression levels of *IL1B* and *ICAM1* in P group palatal gingival tissues extracted by laser microdissection were significantly higher than those in the control group, which were in turn significantly higher than those in the PUM group (Fig 6A). Although there was no significant difference in expression levels between the control and P groups on day 14, expression in the P group remained significantly higher than that in the PUM group (Fig 6B). In addition, the mRNA expression levels of *TNFα* and *RANKL* were eight-fold and three-fold higher in the P group than in the control and PUM groups, respectively, on both days 7 and 14 (Fig 6A and 6B). Conversely, there was no significant difference in expression between the control and PUM groups. In contrast, the buccal gingival tissues showed no significant difference between the P group and PUM group on day 7 (Fig 7A) and day 14 (Fig 7B). In contrast, the relative mRNA expression of *ICAM1* on day 7 in the P group was significantly higher than that in control group (Fig 7A) and that of *TNFα* on day 14 in the P group and PUM group was significantly higher than that in the control groups (Fig 7B).

## Discussion

We previously reported that the ultrasound-microbubble method could be used as an effective tool to transfect NF-κB decoy ODN into rodent periodontal tissues [21]. However, in that previous study, we used only healthy mice as targets. In this study, we applied the ultrasound-microbubble technique to transfect the NF-κB decoy ODN into the gingiva of rats with ligature-induced periodontitis, and then evaluated the effectiveness of this decoy ODN as a potential treatment modality against periodontitis progression. The ligature-induced periodontitis model utilized in this study was consistent with that used in previous studies. For example, Toker et al. induced periodontitis by binding silk sutures around rat molars and reported that plaque and bacteria accumulation increased in the region where the silk sutures contacted the tissue [29]. However, to the best of our knowledge, ours is the first study to evaluate the efficacy of a decoy ODN in treating periodontitis.

To evaluate the effects of decoy ODN-mediated NF-κB suppression, we examined the expression levels of the genes encoding IL-1β, TNF-α, ICAM-1, and RANKL in treated and untreated gingival tissues. Notably, each of these cytokines and cell adhesion molecules is known to play crucial roles in the progression of periodontal disease. Specifically, in alveolar bone, IL-1β is known to stimulate bone resorption and inhibit bone formation [30]. In addition, Stashenko et al. suggested that there is a positive correlation between IL-1β/TNF-α expression in the gingiva and levels of attachment loss and progression of periodontitis [31]. Moreover, it has been revealed that T cells support spontaneous osteoclastogenesis in periodontal patients via RANKL and TNF-α overexpression [32, 33]. Previous studies have shown that RANKL and TNF-α are co-regulated and that TNF-α induces RANKL expression in the gingival epithelium and connective tissue [34, 35]. Lastly, the cell adhesion molecule ICAM-1 is a transmembrane protein that is often expressed in endothelial tissues and leukocytes, and has been shown to play a key role in cell–cell interactions. Furthermore, ICAM-1 appears to facilitate the endothelial transmigration of leukocytes in the initial stage of gingival inflammation [36]. As these essential proteins are all regulated by NF-κB, this transcription factor is thought to be a critical modulator of the progression of periodontitis.

Several studies have reported novel methods for drug/gene delivery into the gingiva, including antimicrobial drug-eluting implants [37], the delivery of naked plasmid DNA with ultrasound and bubble liposomes [38], and decoy ODN transfection via injection into the gingiva dogs [39]. In this study, we sought to determine the feasibility of using the ultrasound-microbubble technique, which was developed by Inagaki et al., for transfection of gingival tissues with the NF-κB decoy ODN [20]. Notably, this method was previously shown to effectively enable the transfection of decoy ODNs, genes, or drugs in a non-invasive, efficient, rapid, and focal manner. Microbubble treatment results in the formation of small holes in cell membranes, which allow the immediate passage of genes, drugs, or in this case, decoy ODN, into the cell cytoplasm [40]; the holes then disappear immediately, resulting in no lasting damage [41]. Our previous research showed that the ultrasound-microbubble technique can be used to successfully deliver decoy ODN to gingival tissues 2 hours after transfection [21].

In this study, we investigated the suppressive effect of ultrasound-microbubble transfected NF-κB decoy ODN on alveolar bone loss caused by periodontitis by measuring the ABC–CEJ distance and the volume of alveolar bone around the maxillary second molar on days 7 and 14 after ligation. In the P group, the ABC–CJE distance between M1 and M2 on day 14 was statistically larger than that on day 7, but the distance between M2 and M3 was statistically equivalent at the two time points. This discrepancy could be due to the pressure or presence of the knot in the silk ligatures preferentially causing alveolar bone loss on the mesial side of M2. In addition, in the histological analysis, inflammatory responses representative of the progression of periodontitis, such as thickening of the junctional epithelium and vasodilation in the gingival connective tissue [42, 43] were effectively suppressed in the PUM group. We also showed overexpression of *IL1B*, *TNFα*, *ICAM1*, and *RANKL* mRNA in the palatal gingival tissues of periodontitis model rats, which is consistent with previous reports [39, 44, 45]. However, on day 14 after ligation, the expression of *ICAM1* in the P group was not higher than that in the control group, in contrast to day 7. Previous research indicated that the expression of *ICAM1* is regulated by certain inflammatory cytokines, including IL-1β and TNF-α [46], and that ICAM-1 plays an important role in the initial stage of periodontitis [47]. Our current results indicate that ICAM-1 is produced and functions during the relatively early period of periodontitis. In contrast, in the PUM group, the expression levels of *IL1B*, *TNFA*, *ICAM1*, and *RANKL* were all lower than those in the P group on both days 7 and 14 after ligation. Based on these results, we conclude that transfection of the NF-κB decoy ODN can suppress the overexpression of the downstream factors IL-1β, TNF-α, and ICAM-1 in the palatal gingival tissues of rats with periodontitis. Interestingly, our results revealed that *RANKL* expression was also suppressed by the decoy ODN, even though RANKL acts upstream of NF-κB. This effect could be due to the fact that downregulation of inflammatory cytokines, including IL-1β and TNF-α, can affect RANKL expression in the periodontium [48]. Additionally, in buccal gingival tissue, there were no significant differences in the expression of *IL1B*, *TNFα*, *ICAM1*, and *RANKL* between the P and PUM groups. The expression of *ICAM1* in the P group on day 7 and that of *TNFα* in the P and PUM groups on day 14 were significantly higher than those in the control group. These remarkable results suggest that the ultrasound-microbubble technique irradiated from the palatal side had limited effective areas and also suggest differences in gingival responses in ligature-induced periodontitis model rats between the palatal and buccal gingiva. Further analysis is necessary to determine the mechanism underlying these results. Therefore, we conclude that the NF-κB decoy ODN is capable of reducing the expression of these inflammatory mediators; as a result, this environment of attenuated inflammation might control RANKL activation, thereby preventing alveolar bone loss. These results demonstrate the usefulness of the ultrasound-microbubble technique as a method for NF-κB decoy ODN transfection into gingival tissues under inflammatory conditions, similar to arterial injury in mice [20]. Moreover, the NF-κB decoy ODN acts in gingival tissues as a suppressor of inflammatory cytokine and cell adherence molecule overexpression, which results from periodontitis. A limitation of this research, however, is that we used a ligation-induced periodontitis model without periodontal pathogens. Further investigation is therefore required to clarify the efficacy of this method in the context of infections with periodontal bacteria before development and application of this novel treatment modality for periodontitis.

In conclusion, we have established a method for transcutaneous transfection of the NF-κB decoy ODN into periodontitis lesions using the ultrasound-microbubble technique. Our findings suggest that this decoy ODN acts as a suppresser of gingival inflammation and of periodontal disease progression.

## Supporting information

S1 Table Body weight. (XLSX)

S2 Table MicroCT analysis. (XLSX)

S3 Table RT-PCR. (XLSX)

## References

[1] Pihlstrom BL, Michalowicz BS, Johnson NW. Periodontal diseases. Lancet. 2005;366(9499):1809–20. doi: 10.1016/S0140-6736(05)67728-8 16298220 10.1016/S0140-6736(05)67728-8

[2] Lindhe J, Westfelt E, Nyman S, Socransky SS, Haffajee AD. Long-term effect of surgical/non-surgical treatment of periodontal disease. J Clin Periodontol. 1984;11(7):448–58. 6378986 10.1111/j.1600-051x.1984.tb01344.x

[3] van Winkelhoff AJ, Rams TE, Slots J. Systemic antibiotic therapy in periodontics. Periodontol 2000. 1996;10:45–78. 9567937 10.1111/j.1600-0757.1996.tb00068.x

[4] Latanich CA, Toledo-Pereyra LH. Searching for NF-kappaB-based treatments of ischemia reperfusion injury. J Invest Surg. 2009;22(4):301–15. 19842907 10.1080/08941930903040155

[5] Sen R, Baltimore D. Inducibility of kappa immunoglobulin enhancer-binding protein Nf-kappa B by a posttranslational mechanism. Cell. 1986;47(6):921–8. 3096580 10.1016/0092-8674(86)90807-x

[6] Tomita N, Morishita R, Tomita S, Gibbons GH, Zhang L, Horiuchi M, et al Transcription factor decoy for NFkappaB inhibits TNF-alpha-induced cytokine and adhesion molecule expression in vivo. Gene Ther. 2000;7(15):1326–32. doi: 10.1038/sj.gt.3301243 10918504 10.1038/sj.gt.3301243

[7] Takayanagi H. Osteoimmunology and the effects of the immune system on bone. Nat Rev Rheumatol. 2009;5(12):667–76. doi: 10.1038/nrrheum.2009.217 19884898 10.1038/nrrheum.2009.217

[8] Morishita R, Tomita N, Kaneda Y, Ogihara T. Molecular therapy to inhibit NFkappaB activation by transcription factor decoy oligonucleotides. Curr Opin Pharmacol. 2004;4(2):139–46. doi: 10.1016/j.coph.2003.10.008 15063357 10.1016/j.coph.2003.10.008

[9] Morishita R, Sugimoto T, Aoki M, Kida I, Tomita N, Moriguchi A, et al In vivo transfection of cis element "decoy" against nuclear factor-kappaB binding site prevents myocardial infarction. Nat Med. 1997;3(8):894–9. 9256281 10.1038/nm0897-894

[10] Kunugiza Y, Tomita T, Tomita N, Morishita R, Yoshikawa H. Inhibitory effect of ribbon-type NF-kappaB decoy oligodeoxynucleotides on osteoclast induction and activity in vitro and in vivo. Arthritis Res Ther. 2006;8(4):R103 doi: 10.1186/ar1980 16813665 10.1186/ar1980 PMC1779370

[11] Nakamura H, Aoki M, Tamai K, Oishi M, Ogihara T, Kaneda Y, et al Prevention and regression of atopic dermatitis by ointment containing NF-kB decoy oligodeoxynucleotides in NC/Nga atopic mouse model. Gene Ther. 2002;9(18):1221–9. doi: 10.1038/sj.gt.3301724 12215889 10.1038/sj.gt.3301724

[12] Egashira K, Suzuki J, Ito H, Aoki M, Isobe M, Morishita R. Long-term follow up of initial clinical cases with NF-kappaB decoy oligodeoxynucleotide transfection at the site of coronary stenting. J Gene Med. 2008;10(7):805–9. doi: 10.1002/jgm.1192 18425985 10.1002/jgm.1192

[13] Fechheimer M, Boylan JF, Parker S, Sisken JE, Patel GL, Zimmer SG. Transfection of mammalian cells with plasmid DNA by scrape loading and sonication loading. Proc Natl Acad Sci U S A. 1987;84(23):8463–7. 2446324 10.1073/pnas.84.23.8463 PMC299564

[14] Paefgen V, Doleschel D, Kiessling F. Evolution of contrast agents for ultrasound imaging and ultrasound-mediated drug delivery. Front Pharmacol. 2015;6:197 Epub 2015/10/07. doi: 10.3389/fphar.2015.00197 26441654 10.3389/fphar.2015.00197 PMC4584939

[15] Leow CH, Iori F, Corbett R, Duncan N, Caro C, Vincent P, et al Microbubble void imaging: a non-invasive technique for flow visualisation and quantification of mixing in large vessels using plane wave ultrasound and controlled microbubble contrast agent destruction. Ultrasound Med Biol. 2015;41(11):2926–37. doi: 10.1016/j.ultrasmedbio.2015.06.023 26297515 10.1016/j.ultrasmedbio.2015.06.023

[16] Stride E, Porter C, Prieto AG, Pankhurst Q. Enhancement of microbubble mediated gene delivery by simultaneous exposure to ultrasonic and magnetic fields. Ultrasound Med Biol. 2009;35(5):861–8. doi: 10.1016/j.ultrasmedbio.2008.11.010 19282096 10.1016/j.ultrasmedbio.2008.11.010

[17] Ferrara K, Pollard R, Borden M. Ultrasound microbubble contrast agents: fundamentals and application to gene and drug delivery. Annu Rev Biomed Eng. 2007;9:415–47. doi: 10.1146/annurev.bioeng.8.061505.095852 17651012 10.1146/annurev.bioeng.8.061505.095852

[18] Taniyama Y, Morishita R. [Development of plasmid DNA-based gene transfer]. Yakugaku Zasshi. 2006;126(11):1039–45. 17077610 10.1248/yakushi.126.1039

[19] Suzuki J, Ogawa M, Takayama K, Taniyama Y, Morishita R, Hirata Y, et al Ultrasound-microbubble-mediated intercellular adhesion molecule-1 small interfering ribonucleic acid transfection attenuates neointimal formation after arterial injury in mice. J Am Coll Cardiol. 2010;55(9):904–13. doi: 10.1016/j.jacc.2009.09.054 20185042 10.1016/j.jacc.2009.09.054

[20] Inagaki H, Suzuki J, Ogawa M, Taniyama Y, Morishita R, Isobe M. Ultrasound-microbubble-mediated NF-kappaB decoy transfection attenuates neointimal formation after arterial injury in mice. J Vasc Res. 2006;43(1):12–8. doi: 10.1159/000089103 16244495 10.1159/000089103

[21] Yamaguchi H, Ishida Y, Hosomichi J, Suzuki JI, Usumi-Fujita R, Shimizu Y, et al A new approach to transfect NF-kappaB decoy oligodeoxynucleotides into the periodontal tissue using the ultrasound-microbubble method. Int J Oral Sci. 2017;9(2):80–86. doi: 10.1038/ijos.2017.10 28452376 10.1038/ijos.2017.10 PMC5518970

[22] Inoue H, Arai Y, Kishida T, Shin-Ya M, Terauchi R, Nakagawa S, et al Sonoporation-mediated transduction of siRNA ameliorated experimental arthritis using 3 MHz pulsed ultrasound. Ultrasonics. 2014;54(3):874–81. doi: 10.1016/j.ultras.2013.10.021 24291002 10.1016/j.ultras.2013.10.021

[23] Nakata T, Umeda M, Masuzaki H, Sawai H. The expression of 11β-hydroxysteroid dehydrogenase type 1 is increased in experimental periodontitis in rats. BMC Oral Health. 2016;16:108 doi: 10.1186/s12903-016-0303-z 27716163 10.1186/s12903-016-0303-z PMC5048409

[24] Wee JH, Zhang YL, Rhee CS, Kim DY. Inhibition of Allergic Response by Intranasal Selective NF-κB Decoy Oligodeoxynucleotides in a Murine Model of Allergic Rhinitis. Allergy Asthema Immnunol Res. 2017;9(1):61–69. 10.4168/aair.2017.9.1.61 PMC5102837 27826963

[25] Cheng WC, Huang RY, Chiang CY, Chen JK, Liu CH, Chu CL, et al Ameliorative effect of quercetin on the destruction caused by experimental periodontitis in rats. J Periodontal Res. 2010;45(6):788–95. doi: 10.1111/j.1600-0765.2010.01301.x 20663021 10.1111/j.1600-0765.2010.01301.x

[26] Hatipoglu M, Saglam M, Koseoglu S, Koksal E, Keles A, Esen HH. The effectiveness of *Crataegus orientalis* M Bieber. (Hawthorn) extract administration in preventing alveolar bone loss in rats with experimental periodontitis. PLoS One. 2015;10(6):e0128134 doi: 10.1371/journal.pone.0128134 26030160 10.1371/journal.pone.0128134 PMC4452266

[27] Vrana JA, Gamez JD, Madden BJ, Theis JD, Bergen HR 3rd, Dogan A. Classification of amyloidosis by laser microdissection and mass spectrometry-based proteomic analysis in clinical biopsy specimens. Blood. 2009;114(24):4957–9. doi: 10.1182/blood-2009-07-230722 19797517 10.1182/blood-2009-07-230722

[28] Livak KJ, Schmittgen TD. Analysis of relative gene expression data using real-time quantitative PCR and the 2(-Delta Delta C(T)) method. Methods (San Diego, Calif). 2001;25(4):402–8. 10.1006/meth.2001.1262 11846609

[29] Toker H, Ozan F, Ozer H, Ozdemir H, Eren K, Yeler H. A morphometric and histopathologic evaluation of the effects of propolis on alveolar bone loss in experimental periodontitis in rats. J Periodontol. 2008;79(6):1089–94. doi: 10.1902/jop.2008.070462 18533788 10.1902/jop.2008.070462

[30] Havemose-Poulsen A, Holmstrup P. Factors affecting IL-1-mediated collagen metabolism by fibroblasts and the pathogenesis of periodontal disease: a review of the literature. Crit Rev Oral Biol Med. 1997;8(2):217–36. 9167094 10.1177/10454411970080020801

[31] Stashenko P, Fujiyoshi P, Obernesser MS, Prostak L, Haffajee AD, Socransky SS. Levels of interleukin 1 beta in tissue from sites of active periodontal disease. J Clin Periodontol. 1991;18(7):548–54. 1894750 10.1111/j.1600-051x.1991.tb00088.x

[32] Brunetti G, Colucci S, Pignataro P, Coricciati M, Mori G, Cirulli N, et al T cells support osteoclastogenesis in an in vitro model derived from human periodontitis patients. J Periodontol. 2005;76(10):1675–80. doi: 10.1902/jop.2005.76.10.1675 16253089 10.1902/jop.2005.76.10.1675

[33] Taubman MA, Valverde P, Han X, Kawai T. Immune response: the key to bone resorption in periodontal disease. J Periodontol. 2005;76(11 Suppl):2033–41. doi: 10.1902/jop.2005.76.11-S.2033 16277573 10.1902/jop.2005.76.11-S.2033

[34] Fujihara R, Usui M, Yamamoto G, Nishii K, Tsukamoto Y, Okamatsu Y, et al Tumor necrosis factor-alpha enhances RANKL expression in gingival epithelial cells via protein kinase A signaling. J Periodontal Res. 2014;49(4):508–17. doi: 10.1111/jre.12131 24102429 10.1111/jre.12131

[35] Belibasakis GN, Bostanci N, Hashim A, Johansson A, Aduse-Opoku J, Curtis MA, et al Regulation of RANKL and OPG gene expression in human gingival fibroblasts and periodontal ligament cells by *Porphyromonas gingivalis*: a putative role of the Arg-gingipains. Microbial pathogenesis. 2007;43(1):46–53. doi: 10.1016/j.micpath.2007.03.001 17448630 10.1016/j.micpath.2007.03.001

[36] Erdemir EO, Hendek MK, Keceli HG, Apan TZ. Crevicular fluid levels of interleukin-8, interleukin-17 and soluble intercellular adhesion molecule-1 after regenerative periodontal therapy. Eur J Dent. 2015;9(1):60–5. doi: 10.4103/1305-7456.149644 25713486 10.4103/1305-7456.149644 PMC4319302

[37] Lee FY, Chen DW, Hu CC, Hsieh YT, Liu SJ, Chan EC. In vitro and in vivo investigation of drug-eluting implants for the treatment of periodontal disease. AAPS PharmSciTech. 2011;12(4):1110–5. doi: 10.1208/s12249-011-9681-3 21879391 10.1208/s12249-011-9681-3 PMC3225558

[38] Sugano M, Negishi Y, Endo-Takahashi Y, Suzuki R, Maruyama K, Yamamoto M, et al Gene delivery system involving Bubble liposomes and ultrasound for the efficient in vivo delivery of genes into mouse tongue tissue. Int J Pharm. 2012;422(1–2):332–7. doi: 10.1016/j.ijpharm.2011.11.001 22100513 10.1016/j.ijpharm.2011.11.001

[39] Shimizu H, Nakagami H, Morita S, Tsukamoto I, Osako MK, Nakagami F, et al New treatment of periodontal diseases by using NF-kappaB decoy oligodeoxynucleotides via prevention of bone resorption and promotion of wound healing. Antioxid Redox Signal. 2009;11(9):2065–75. doi: 10.1089/ARS.2008.2355 19186992 10.1089/ars.2008.2355

[40] Taniyama Y, Tachibana K, Hiraoka K, Namba T, Yamasaki K, Hashiya N, et al Local delivery of plasmid DNA into rat carotid artery using ultrasound. Circulation. 2002;105(10):1233–9. 11889019 10.1161/hc1002.105228

[41] Endoh M, Koibuchi N, Sato M, Morishita R, Kanzaki T, Murata Y, et al Fetal gene transfer by intrauterine injection with microbubble-enhanced ultrasound. Mol Ther. 2002;5(5 Pt 1):501–8. doi: 10.1006/mthe.2002.0577 11991740 10.1006/mthe.2002.0577

[42] Bosshardt DD, Lang NP. The Junctional epithelium: from health to disease. J Dent Res. 2005;84(1):9–20. doi: 10.1177/154405910508400102 15615869 10.1177/154405910508400102

[43] Kornman KS, Page RC, Tonetti MS. The host response to the microbial challenge in periodontitis: assembling the players. Periodontol 2000. 1997;14:33–53. 9567965 10.1111/j.1600-0757.1997.tb00191.x

[44] Lu SH, Huang RY, Chou TC. Magnolol ameliorates ligature-induced periodontitis in rats and osteoclastogenesis: in vivo and in vitro study. Evid Based Complement Alternat Med. 2013;2013:634095 doi: 10.1155/2013/634095 23573141 10.1155/2013/634095 PMC3618931

[45] Xu XC, Chen H, Zhang X, Zhai ZJ, Liu XQ, Zheng XY, et al Effects of oestrogen deficiency on the alveolar bone of rats with experimental periodontitis. Mol Med Rep. 2015;12(3):3494–502. 26035209 10.3892/mmr.2015.3875 PMC4526094

[46] Shirasaki H, Watanabe K, Kanaizumi E, Sato J, Konno N, Narita S, et al Effect of glucocorticosteroids on tumour necrosis factor-alpha-induced intercellular adhesion molecule-1 expression in cultured primary human nasal epithelial cells. Clin Exp Allergy. 2004;34(6):945–51. doi: 10.1111/j.1365-2222.2004.01964.x 15196284 10.1111/j.1365-2222.2004.01964.x

[47] Tamai R, Asai Y, Ogawa T. Requirement for intercellular adhesion molecule 1 and caveolae in invasion of human oral epithelial cells by *Porphyromonas gingivalis*. Infection and Immunity. 2005;73(10):6290–8. doi: 10.1128/IAI.73.10.6290-6298.2005 16177300 10.1128/IAI.73.10.6290-6298.2005 PMC1230918

[48] Fujihara R, Usui M, Yamamoto G, Nishi K, Tsukamoto Y, Okamatsu Y, et al Tumor necrosis factor-α enhances RANKL expression in gingival epithelial cells via protein kinase A signaling. 2013;49(4):508–517. 10.1111/jre.12131 24102429

